# A LysM Domain-Containing Gene *OsEMSA1* Involved in Embryo sac Development in Rice (*Oryza sativa* L.)

**DOI:** 10.3389/fpls.2017.01596

**Published:** 2017-09-20

**Authors:** Qian Zhu, Xiao-Ling Zhang, Sadia Nadir, Wen-Hua DongChen, Xiao-Qiong Guo, Hui-Xin Zhang, Cheng-Yun Li, Li-Juan Chen, Dong-Sun Lee

**Affiliations:** ^1^Rice Research Institute, Yunnan Agricultural University Kunming, China; ^2^Department of Chemistry, University of Science and Technology Bannu, Pakistan; ^3^State Key Laboratory for Conservation and Utilization of Bio-Resources in Yunnan, Yunnan Agricultural University Kunming, China; ^4^Key Laboratory for Agricultural Biodiversity and Pest Management of China Education Ministry, Yunnan Agricultural University Kunming, China

**Keywords:** rice (*Oryza sativa* L.), *OsEMSA1*, female gametophyte, embryo sac development, LysM domain

## Abstract

The embryo sac plays a vital role in sexual reproduction of angiosperms. LysM domain containing proteins with multiple lysin motifs are widespread proteins and are involved in plant defense responses against fungal chitins and bacterial peptidoglycans. Various studies have reported the role of LysM domain-containing proteins in plant defense mechanisms but their involvement in sexual reproduction remains largely unknown. Here, we report the involvement of a LysM domain-containing gene, *EMBRYO SAC 1* (*OsEMSA1*), in the sexual reproduction of rice. The gene encoded a LysM domain-containing protein that was necessary for embryo sac development and function. The gene was expressed in root, stem, leaf tissues, panicle and ovaries and had some putative role in hormone regulation. Suppression of *OsEMSA1* expression resulted in a defective embryo sac with poor differentiation of gametophytic cells, which consequently failed to attract pollen tubes and so reduced the panicle seed-setting rate. Our data offers new insight into the functions of LysM domain-containing proteins in rice.

## Introduction

Sexual reproduction in plants begins with gametogenesis. Female gametogenesis is a biphasic process involving complex, tightly orchestrated developmental mechanisms, sequential cell divisions, subsequent nuclei migration, cellularization, and programmed cell death leading to the development of the megagametophyte known as the embryo sac (Reiser and Fischer, 1993; Drews et al., 1998). Female gametophyte development and function depends on the activities of many genes expressed either within itself or in the surrounding cells (Drews et al., 1998; Pagnussat et al., 2005). The female gametophyte, or embryo sac, develops coordinately with the sporophytic tissues of the ovule, making it an ideal model for the study of fundamental processes crucial to development (Drews et al., 1998). The embryo sac is considered pivotal in ensuring successful fertilization, embryogenesis and subsequent seed development (Pagnussat et al., 2005). In several previous studies, the role of phytohormones in controlling the female gametophyte has been well established (Deng et al., 2010; Bencivenga et al., 2012; Cheng et al., 2013). It was reported that manipulation of concentration of the phytohormone auxin results in defects of female gametophyte development (Pagnussat et al., 2009). Not only localized auxin biosynthesis but also auxin import are required for cellularization during embryo sac development (Panoli et al., 2015). Cytokinin was found to be indispensable for the male and female gamete development in *Arabidopsis* (Hirano et al., 2008; Kinoshita-Tsujimura and Kakimoto, 2011; Yamaki et al., 2011; Cheng et al., 2013). The regulatory interaction between auxin and cytokinin has also been linked to the development of the female gametophyte (Schaller et al., 2015).

The ability of the embryo sac to attract a pollen tube is crucial for effective fertilization. During fertilization, the pollen tube grows directionally inside the pistil and delivers the sperm to the embryo sac. The female sporophytic tissues facilitates the pollen tube's journey by providing multiple, stage specific, guidance signals along the pollen tube path (Wang et al., 1993; Cheung et al., 1995; Hulskamp et al., 1995; Ray et al., 1997; Fiebig et al., 2000; Mollet et al., 2000; Wu et al., 2000; Palanivelu et al., 2003; Palanivelu and Tsukamoto, 2012). The final phases of pollen tube guidance are controlled by the embryo sac (Higashiyama et al., 1998). The two synergid cells located in the embryo sac are supposed to secrete some chemotropic substances that attracts the pollen tube (Higashiyama et al., 2001). Recently, it was identified that several cell-surface receptors located on the pollen tube mediates the male perception of female attractant in *Arabidopsis* (Takeuchi and Higashiyama, 2016; Wang et al., 2016). The chemotropic substances secreted by synergids have been identified to be defensin-like LURE peptides (Okuda et al., 2009). Despite the importance of male-female communication in fertilization and subsequently seed and/or fruit production, this phenomenon is poorly understood in rice. As one of the most important crops and a model monocotyledonous plant, various stigma, pistil and pollen-specific, genes have been identified in the rice genome (Yoshida et al., 2005; Park et al., 2006; Li et al., 2007).

LysM are a family of carbohydrate-binding modules with multiple lysin motifs and are widely present in microbes, plants and animals (Shi et al., 2013). The multiple LysMs present in LysM-domain are separated from each other by some short spacer sequences (Buist et al., 2008). LysMs can be found at the end terminals or in the center of a protein (Buist et al., 2008). LysM containing proteins can be secreted proteins, membrane proteins, outer-membrane proteins, lipoproteins or cell wall bound proteins. LysM containing proteins bind to N-acetylglucosamine-containing carbohydrates, such as chitin, chitio-oligosaccharides and peptidoglycanwith various specificities (Akcapinar et al., 2015). Most of the LysM domain containing plant proteins belong to the class of RLKs (receptor like kinases) in plants (Wan, 2015). RLKs have a major role in cellular signaling in plants and most of the LysM domain-containing RLKs are involved in plant-microbial interactions leading to pathogen defense, symbiosis or suppression of host defense (Gust et al., 2012; Tanaka et al., 2013; Wan, 2015). Rice LysM protein CEBiP (chitin elicitor-binding protein) was shown to be involved in chitin recognition and activation of plant innate immunity against chitin (Kaku et al., 2006). The CEBiP protein has an extracellular domain that contains two LysMs. Another protein, OsCERK1 (chitin elicitor receptor kinase 1) function together with CEBiP and is involved in chitin triggered immunity in rice (Shimizu et al., 2010). Two dual acting lysM proteins, OsLYP4 and OsLYP6, were also found to be involved in perception and recognition of bacterial peptidoglycan and fungal chitin (Liu et al., 2012). Silencing of CEBiP, OsCERK1, LYP4, and LYP6 substantially increase susceptibility of *O.sativa* to microbial pathogenesis (Kaku et al., 2006; Shimizu et al., 2010; Liu et al., 2012). The LysM-containing receptor-like kinase1/chitin elicitor receptor kinase1 (LYK1/CERK1) of *Arabidopsis* was shown to be essential for chitin recognition (Miya et al., 2007; Wan et al., 2008; Willmann et al., 2011). Various studies have mentioned the role of LysM domain-containing proteins in plant innate immunity but there has been no report of their role on plant sexual reproduction.

Our previous study identified a rice B_*sister*_-MADS Box gene, *FEMALE-STERILE* (*FST*), which is expressed in the sporophytic tissues of ovules and plays vital roles during ovule and early seed development (Lee et al., 2013). Based on the microarray data of the *fst* mutant, we selected the candidate gene *OsEMSA1* (BankIt1920511, KX503265), a LysM domain-containing gene, located on chromosome 10, whose expression level was significantly down regulated at meiotic stage in rice panicles. This study aimed to explore the role and function of *OsEMSA1* during sexual reproduction in rice. Our results showed that *OsEMSA1* encoded a LysM domain-containing protein that was crucial for embryo sac development in rice. Our findings revealed an important role for a LysM domain-containing protein during sexual reproduction in rice.

## Materials and methods

### Plant material and growth condition

The wild type rice (*O. sativa* ssp. *japonica* cv. Ilmibyeo) was used for rice transformation in this study. Rice seeds were germinated in distilled water and grown in a greenhouse experiment field under natural growing conditions.

### Gene cloning, characterization, and bioinformatic analysis

A 952-bp *OsEMSA1* cDNA fragment was amplified from KOME clone by specific primers OsEMSA1-1F and OsEMSA1-1R (Supplementary Table 2). About 2.2-kb putative promoter upstream of the *OsEMSA1* coding region fragment was amplified by PCR with primers OsEMSA1P-F and OsEMSA1P-R using Ilmibyeo genomic DNA as a template (Supplementary Table 2). Protein sequence and homology analysis was performed using NCBI databases. Protein sequence motifs were identified using the SMART program. Sequence alignments and the phylogenetic tree were constructed using MEGA6 and the neighbor-joining method.

### Binary vector constructs and transgenic plant development

To investigate the expression pattern of *OsEMSA1*, its promoter fragment was fused to the *GUS* reporter gene and subcloned into the binary vector DTV1 (the modified pCAMBIA1305.2 without enhancer) to yield the *pOsEMSA1::GUS* construct. To construct the *OsEMSA1* RNAi vector (*pCaMV35S*×*2::OsEMSA1-RNAi*), a 117-bp intron fragment was used as a linker between a 170-bp gene-specific fragment (a 422-bp gene-specific fragment was generated synchronously) in the antisense and sense orientations. These reconstructed fragments were inserted into the DTV6 binary vector containing a double 35S promoter.

To investigate whether *Os.51835* and *Os.43929* are involved in hormone regulation, *Os.51835* and *Os.43929* RNAi binary vectors (*pCaMV35S*×*2::Os51835-RNAi and pCaMV35S*×*2::Os43929-RNAi*) were constructed, respectively.

All the constructs were introduced into *Agrobacterium tumefaciens* strain EHA105 and subsequently introduced into Ilmibyeo rice embryonic callus. Various T_1_ transgenic plants were generated: 4 lines (each line 10 plants) of *pOsEMSA1::GUS* transgenic plants, 8 lines (each line 10 plants) *pCaMV35S*×*2::OsEMSA1-RNAi1* transgenic plants, 7 lines (each line 10 plants) *pCaMV35S*×*2::OsEMSA1-RNAi2* transgenic plants, 6 lines (each line 10 plants) *pCaMV35S*×*2::Os51835-RNAi* T_1_ transgenic plants and 8 lines (each line 10 plants) *pCaMV35S*×*2::Os43929-RNAi* T_1_ transgenic plants were generated.

### Pollen viability and germination assay

To study pollen viability, spikelets were fixed in Carnoy's fixative solution (99% ethanol: chloroform: glacial acetic acid of 6:3:1) and stained in I_2_-KI and simplified Alexander's staining solution as mentioned previously (Peterson et al., 2010; Wang et al., 2012). Pollen grains from anthers were placed in 1% I_2_-KI staining solution and 1% simplified Alexander's staining solution for 20 min at room temperature to stain the pollen. Pollen grains that were round and stained black by I_2_-KI solution were considered fertile. Pollen grains that were stained purple or red by simplified Alexander's staining solution were considered viable.

Pollen germination and pollen tube growth were examined using aniline blue staining. Spikelets were collected during 30–60 min after flowering and then immediately placed in fixative solution (99% ethanol: glacial acetic acid of 3:1). The fixed sample was hydrated by passing through an ethanol series (70, 50, and 30% and distilled water) for a duration of 10 min for each step at room temperature. Pistils were excised, softened with 1 M NaOH at 60°C for 1 h and subsequently rinsed twice with distilled water, each for 10 min. Pollen tubes were stained with 0.1% (w/v) aniline blue in 100 mM K_3_PO_4_ buffer (pH 11) for 10 min in darkness. Samples were then visualized by UV microscopy (Mori et al., 2006).

### Endogenous hormone assay

Endogenous contents of GA_3_ and IAA were determined using an ELISA hormone assay kit. The fresh tissues including root, stem, leaf and panicle were sampled at flowering stage and finely homogenized in 0.01 M PBS buffer (pH 7.4). The homogenate was centrifuged at 3,000 rpm for 5 min and the supernatant collected and preserved at −70°C. ELISA was performed as described in the protocol provided by the supplier (Plant Hormone Elisa Kit, Colorful Gene Biotechnology Co. Ltd., Wuhan, China). ELISA plates were stored at −20°C and the other reagents at 4°C. The developed plates were analyzed by an automatic microplate reader (Thermo MultiskanMK3, Thermo Fisher) and the average of three readings used from the evaluation of absorbance at 450 nm.

### Paraffin sections of embryo sac analysis

To analyze the embryo sac development, several flowers were selected just before pollination (florets were sampled just before pollination). The ovaries were removed and immediately fixed in cold GA-PFA solution which contained 2.5% glutaraldehyde, 2% paraformaldehyde and 50 mM PIPES (pH 7.2), at 4°C overnight as described previously (Sambrook et al., 1989). The ovaries were removed, dehydrated in a graded ethanol series (30, 50, 70, 80, 90, 95, and 100% [v/v]), with 10 min for each gradient and then embedded in paraffin. Semi thin sections (7 μm) of the embedded organ were cut with a microtome and stained with toluidine blue O. The embryo sac size was measured by Leica microscope software.

### Laser scanning confocal microscopy assay

Whole-mount eosin B staining was performed using laser scanning confocal microscopy to determine embryo sac development (Zhang et al., 2003). The sample fixation method employed is described in the paraffin sectioning above. The ovaries were dissected in 70% ethanol and rehydrated sequentially in 50% ethanol, 30% ethanol and distilled water. Subsequently, the samples were pretreated in 2% KAl(SO_4_)_2_·12H_2_O for 20 min and then stained with 10 mg/l eosin B (C_20_H_6_N_2_O_9_Br_2_Na_2_) in 4% sucrose solution for 10–12 h at room temperature. The samples were post-treated in 2% KAl(SO_4_)_2_·12H_2_O for 20 min, rinsed with distilled water three times and afterwards dehydrated with a series of ethanol solutions: 30, 50, 70, 90, and 100% (v/v). The dehydrated samples were treated in a mixture of absolute ethanol and methyl salicylate (1:1 [v/v]) for 1 h, and then cleared in 100% methyl salicylate solution. The cleared samples were scanned with a Leica laser scanning confocal microscope. Excitation wavelength was 543 nm, and emission light was detected between 550 and 630 nm.

### GUS staining assay

The GUS assay was conducted according to Lee et al. (2013). Tissue samples from *pOsEMSA1::GUS* transgenic plants were immersed in cold 90% acetone at −20°C for 20 min, then rinsed three times with rinse solution: 0.1 M K_3_Fe(CN)_6_, 0.1 M K_4_Fe(CN)_6_, and 0.5 M NaPO_4_, pH 7.2. Samples were soaked in GUS staining solution (10% Triton X-100, 20 mM X-Gluc, 0.1 M K_3_Fe(CN)_6_, 0.1 M K_4_Fe(CN)_6_ and 0.5 M NaPO_4_, pH 7.2) and incubated at 37°C overnight. After staining, the samples were bleached with 75% ethanol and observed under a dissecting microscope.

### Gene expression analysis by RT-PCR

Extraction of total RNA from plant tissues at different developmental stages was performed using the TRNzol reagent (TRNzol, TianGen Biotech Co. Ltd., Beijing, China). The cDNAs were synthesized from 2 mg of total RNA according to the manufacturer's protocol (RevertAid First Strand cDNA Synthesis Kit, Thermo Fisher). Rice β*-ACTIN* was amplified and used as an internal standard to normalize the expression of tested genes. Six pairs of primers were used for RT-PCR (Supplementary Table 2).

### Phenotype characterization

Pollen fertility was calculated by determining the percentage of normal pollen grains against total pollen grains per spikelet. Seed fertility was the number of filled grains divided by the total number of grains per panicle evaluated for each panicle on five plants. Panicle length was measured as the average value in centimeters, from the panicle neck to the panicle tip based on an evaluation of three panicles from 10 random plants. Mature rice seeds were air-dried and stored at room temperature. Fully filled grains were used for grain length, width and weight measurement. Ten randomly chosen grains from each plant were lined up length-wise along a Vernier caliper to measure grain length, and then arranged by breadth to measure grain width. Grain weight was calculated based on 100 grains and converted to thousand-grain weight.

## Results

### *OsEMSA1* cloning and characterization

**Figure 1:**
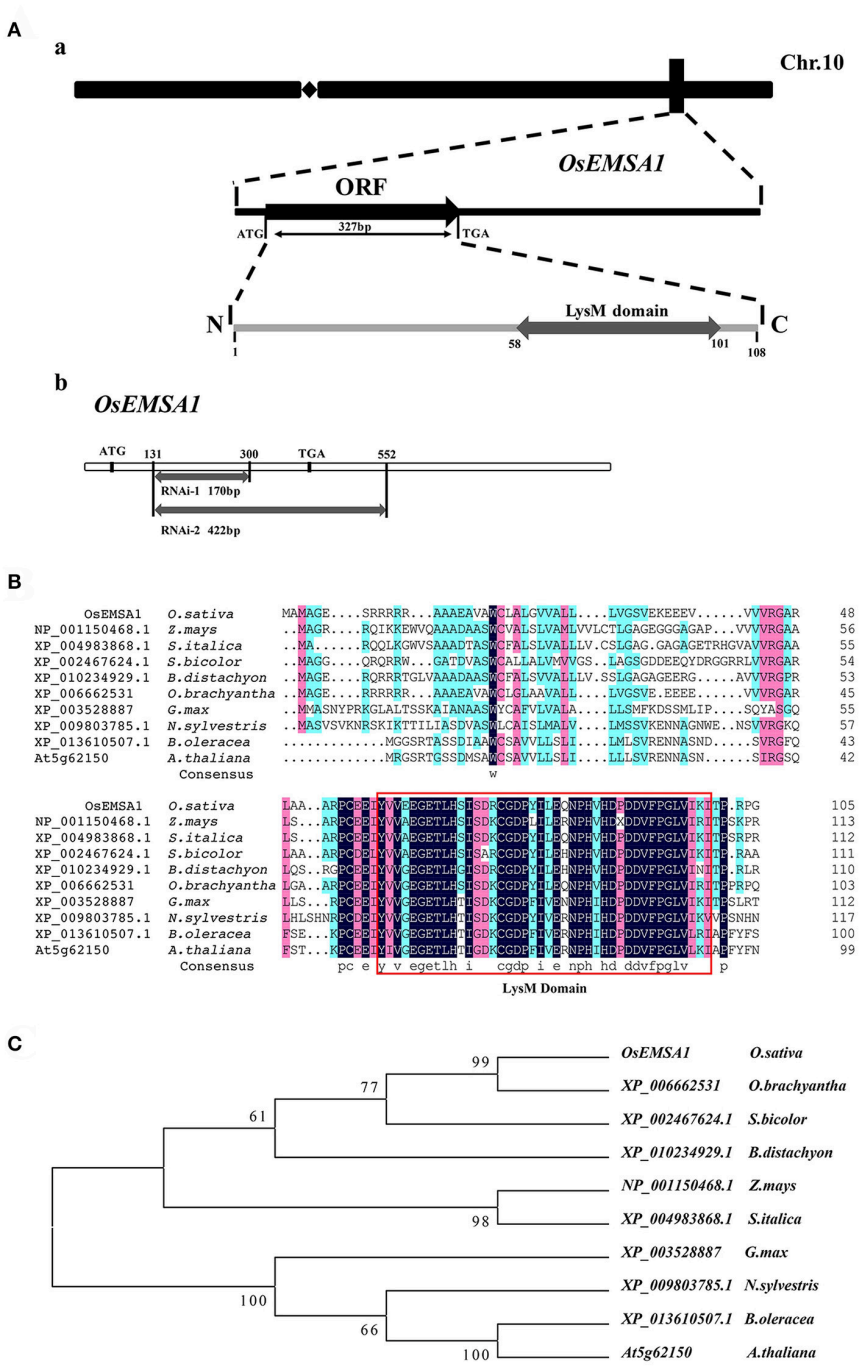
Molecular cloning and phylogenetic analysis. **(A)** Diagram of the *OsEMSA1*. **(a)** *OsEMSA1* was located on chromosome 10. ORF is shown as a black box and the LysM domain as a gray box. **(b)** Two different fragments for RNAi construction are shown as gray boxes. **(B)** Sequence alignment of OsEMSA1 protein and its homologs in plants, generated with DNAMAN. The LysM domain is outlined with the red box. **(C)** Phylogenetic analysis of OsEMSA1 protein and its homologs in plants based on the alignment above. The phylogenetic tree was constructed by neighbor-joining method. Bootstrap value = 1,000.

The *OsEMSA1* gene was cloned to allow exploring its function. We isolated the 2.6 kb putative promoter region of *OsEMSA1* and the 952-bp full-length cDNA (GenBank accession number: KX503265) with 327 bp open reading frame (ORF), which encodes a protein with 108 amino acid residues (Figure 1Aa). The protein domain identification tool, SMART, identified an N-terminal signal peptide at position 1–37 and a LysM domain at the C-terminal at position 58–101 (Figures 1A,B). A search on the Protein BLAST NCBI database identified a number of homologs with a conserved LysM domain. Phylogenetic analysis showed that the protein is distributed in monocotyledons and dicotyledons but with no specific function assigned (Figure 1C). To investigate potential regulatory *cis*-acting elements, we analyzed the promoter region of *OsEMSA1* using PlantCARE—this detailed analysis revealed that it contained 24 different cis-regulatory elements involved in various processes. In addition to the typical TATA-box and CAAT-box, there were hormone responsive elements, growth regulators, metabolism regulators and several stress-responsive regulatory elements found (Supplementary Table 1). Most of these predicted elements are involved in growth and stress responses, suggesting that the OsEMSA1 promoter may play multi-functional roles.

### Expression pattern of *OsEMSA1*

**Figure 2:**
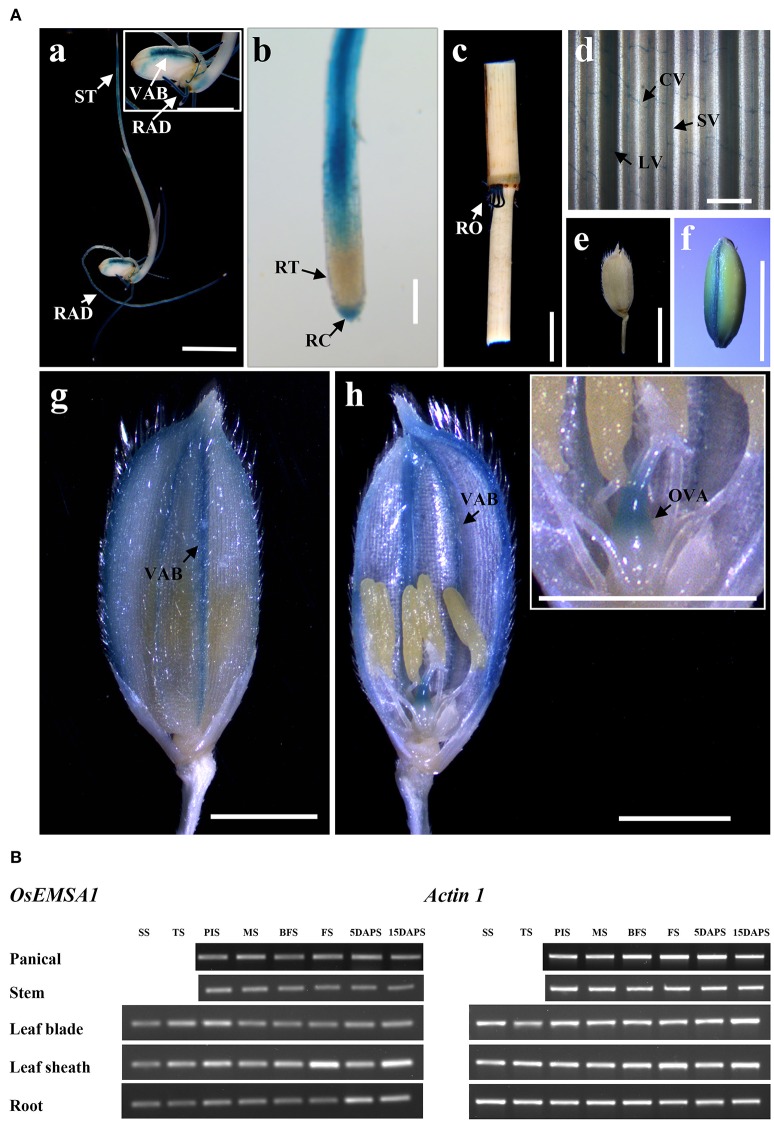
Expression pattern of *OsEMSA1*. **(A)** *OsEMSA1* expression pattern in different tissues of *pOsEMSA1::GUS* transgenic rice. **(a,f)** GUS expression in the shoot tip, radicle and dorsal vascular bundle. **(b)** Elongation zone of radicle and root cap. **(c)** Mature root. **(d)** Commissural vein in leaf. **(e)** Mature spikelet. **(g,h)** Ovary and vascular bundles of glume. RAD, radicle; ST, shoot tip; VAB, vascular bundle; RT, root tip; RC, root cap; RO, root; CV, commissural vein; LV, large vein; SV, small vein; OVA, ovary. Bars = 5 mm in (**a,c,e,f)**; 2 mm in **(g,h)**. **(B)** RT-PCR analysis of *OsEMSA1*. SS, seedling stage; TS, tillering stage; PIS, panicle initiation stage; MS, meiotic stage; BFS, before flowering stage; FS, flowering stage; 5DAPS, 5 days after pollination stage; 15DAPS, 15 days after pollination stage.

Semi quantitative RT-PCR analyses using total RNA samples from various tissues at different developmental stages were performed to determine the *OsEMSA1* expression profile. Different levels of transcription was detected in various organs from early vegetative to reproductive stages. Expression of *OsEMSA1* was detected before pollination to 15 days after pollination. High expression was observed in roots during all developmental stages. Expression of the gene was also observed in the panicle, stem, leaf sheath, leaf blade and at the panicle initiation stage (Figure 2B). To better understand the *OsEMSA1* expression pattern, the *OsEMSA1* promoter was fused to the *GUS* reporter gene and introduced into wild type rice Ilmibyeo by *Agrobacterium-*mediated transformation approach. Histochemical staining of GUS showed high expression of *OsEMSA1* in various tissues at different developmental stages. In consonance with RT-PCR results in roots, there was very high activity of *OsEMSA1* in roots at all developmental stages. GUS activity in roots was found in the root elongation zone, lateral roots and vascular bundle (Figures 2Aa–c). GUS activity was also observed in the commissural vein of leaves and spikelet after heading stage (Figures 2Ad,g). Notably, in female reproductive organs, GUS activity was high at the early stages of flowering. Results showed that *OsEMSA1* was mainly expressed in the ovary (Figure 2Ah).

### *OsEMSA1* does not affect vegetative growth and pollen development

**Figure 3:**
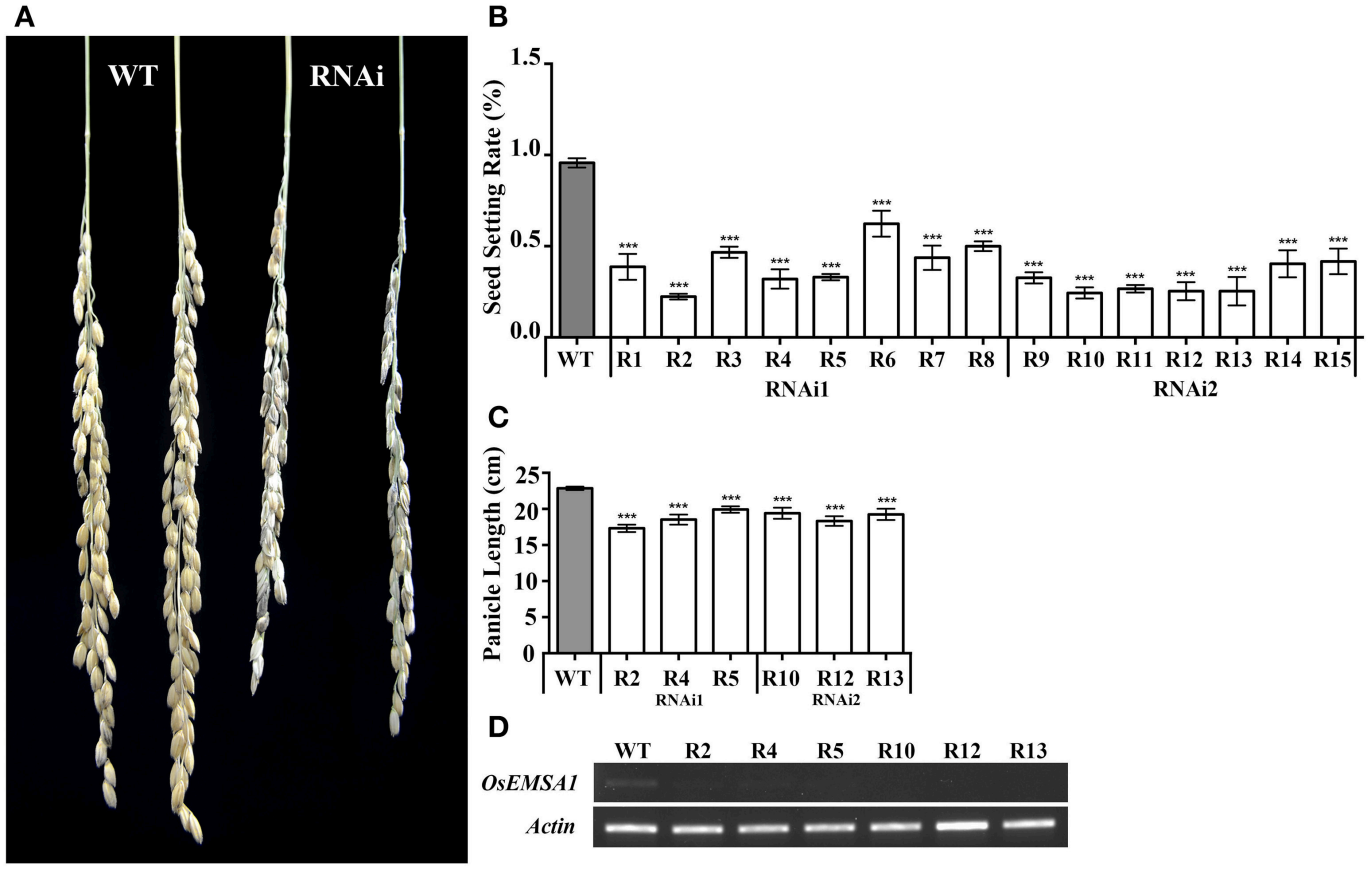
Biological trait comparisons of WT and *OsEMSA1* RNAi lines. **(A)** Panicles of WT and *OsEMSA1* RNAi plants. R1 to R15 are all *OsEMSA1* RNAi lines. **(B)** Seed setting rate of T_1_ *OsEMSA1* RNAi lines. **(C)** Panicle length of candidate *OsEMSA1* RNAi lines. **(D)** *OsEMSA1* expression analysis in RNAi lines. The whole-plant RNA samples were used in RT-PCR. Values are mean ± SD. Asterisks indicated significant differences (^*^P < 0.05) and extremely significant differences (^**^P < 0.01 and ^***^P < 0.001).

To determine whether *OsEMSA1* regulates growth or reproductive processes, we generated two types of *OsEMSA1* RNAi (*pCaMV35S*×*2::OsEMSA1-RNAi1* and *pCaMV35S*×*2::OsEMSA1-RNAi2*) transgenic plants (Figure 1Ab). A significant number of RNAi transgene events were associated with completely failed sexual reproduction, and thus, could not be characterized in detail. Only those transgenic plants where reproduction succeeded at some level were selected for further characterization. The transgenic plants exhibited normal vegetative growth patterns in terms of germination, tillering and elongation. However, the overall seed-setting rate of both types of the RNAi lines was significantly decreased as compared to the wild-type (WT) (Figure 3B). Among these transgenic plants, lines R2, R4, R5, R10, R12, and R13 whose seed setting were significantly arrested, were selected as representatives for further analysis. The expression of *OsEMSA1* was significantly down-regulated in these RNAi lines (Figure 3D). Panicle lengths of these transgenic lines were examined and were found to be 4 cm shorter (18.23 ± 0.66 to 22.85 ± 0.24 cm) than the WT (Figures 3A,C).

**Figure 4:**
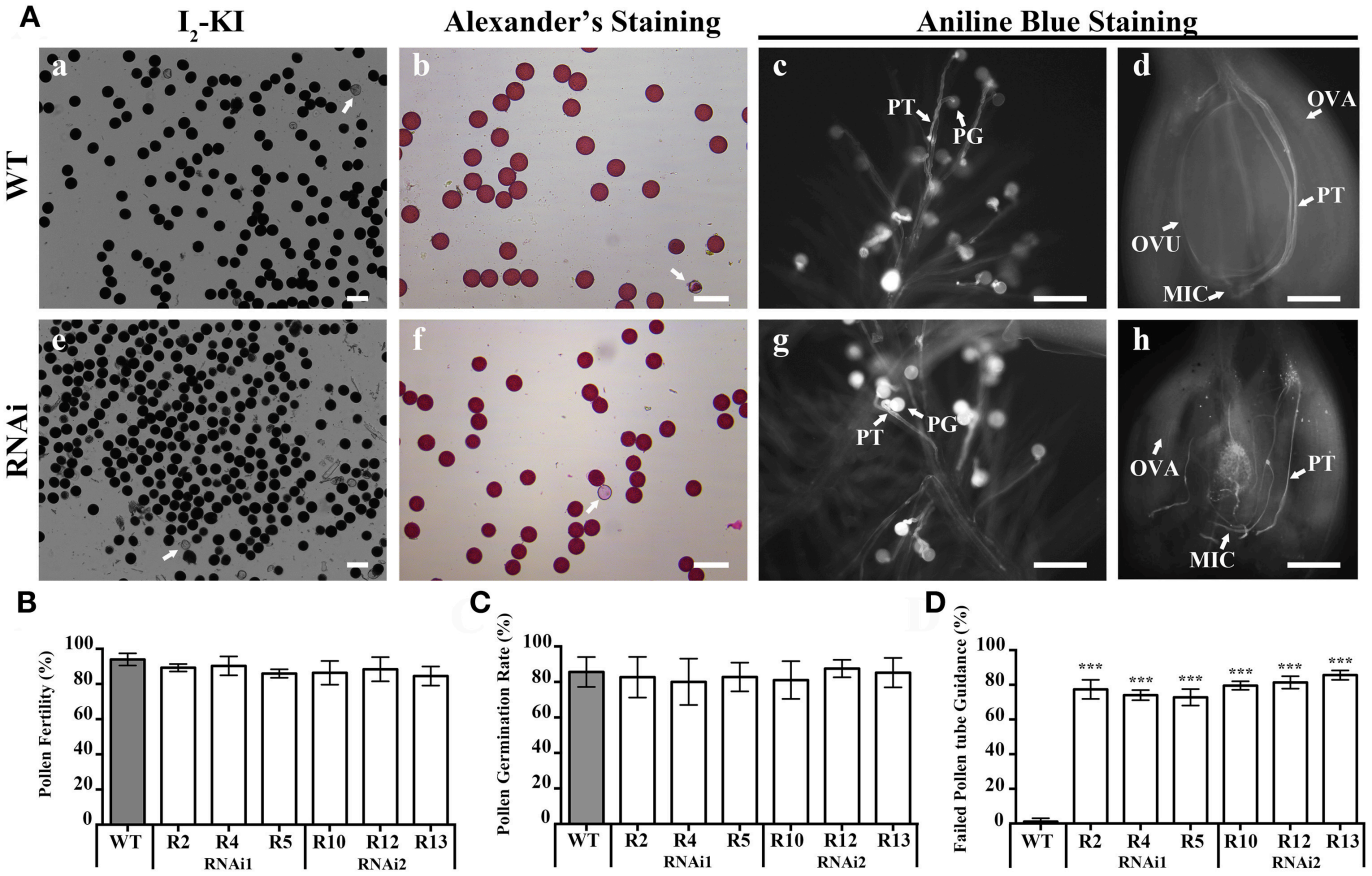
Pollen viability and pollen germination in WT and *OsEMSA1* RNAi lines. **(A)** Pollen viability assay by I_2_-KI and Alexander's staining. Pollen germination assay by Aniline blue staining. **(a–d)** WT pollen grain and ovary. **(e–h)** RNAi plant pollen grain and ovary. Arrows indicate aborted pollen grain in **(a,b,e,f)**. WT, wild-type; PG, pollen grain; PT, pollen tube; OVA, ovary; OVU, ovule; MIC, micropyle. Bars = 100 μm. **(B)** Percentage of viable pollen grain in WT plants and RNAi plants. **(C)** Pollen germination rate in WT plants and RNAi plants. **(D)** Percentage of random path pollen tube in WT plants and RNAi plants. Values are mean ± SD. Asterisks indicated extremely significant differences (^***^P < 0.001).

The overall decrease in seed setting rate indicated an ineffective fertilization. Effective fertilization requires functional male and female gametophytes that produce functional sex gametes. To explore the role of *OsEMSA1* in male gametogenesis, we examined the development of male reproductive organs. Pollen from the representative RNAi transgenic lines and WT were examined by iodine-potassium iodide (I_2_-KI) and simplified Alexander's stain methods to determine fertility and viability, respectively (Figures 4Aa,b; Figures 4Ae,f). Statistical analysis showed that viability rate of pollens in RNAi lines were not different from that of WT (Figure 4B). We further determined the pollen germination and pollen tube growth in the transgenic lines (Figures 4Ac,d; Figures 4Ag,h). Consistent to WT, approximately 80–85% pollen from the transgenic lines germinated and produced pollen tubes with no morphological difference to the WT (Figure 4C).

### *OsEMSA1* regulates female gametophyte development

Interestingly, Pollen tubes germinated and grew normally in RNAi plants. Pollen tubes entered the pistil through the stigmatic cells and reached the ovules similarly to that of the WT, indicating normal pollen tube growth and sporophytic guidance. However, within the ovary, pollen tubes of the RNAi lines behaved differently. Pollen tubes of the RNAi lines grew toward the ovule but instead of entering the micropylar opening, 80% of them seemed to lose their path and coiled randomly (Figures 4Ah,D). In some cases, pollen tubes were found to wrap around the ovules. The pollen tubes reached the micropylar end but could not enter the embryo sac, indicating that pollen tube guidance was not normal and that the RNAi lines failed to attract pollen tubes. However, pollen tubes of the WT successfully entered the female gametophyte (Figure 4Ad). Together, these results indicated that the mutation in *OsEMSA1* did not affect the development and function of the male gametophyte.

**Figure 5:**
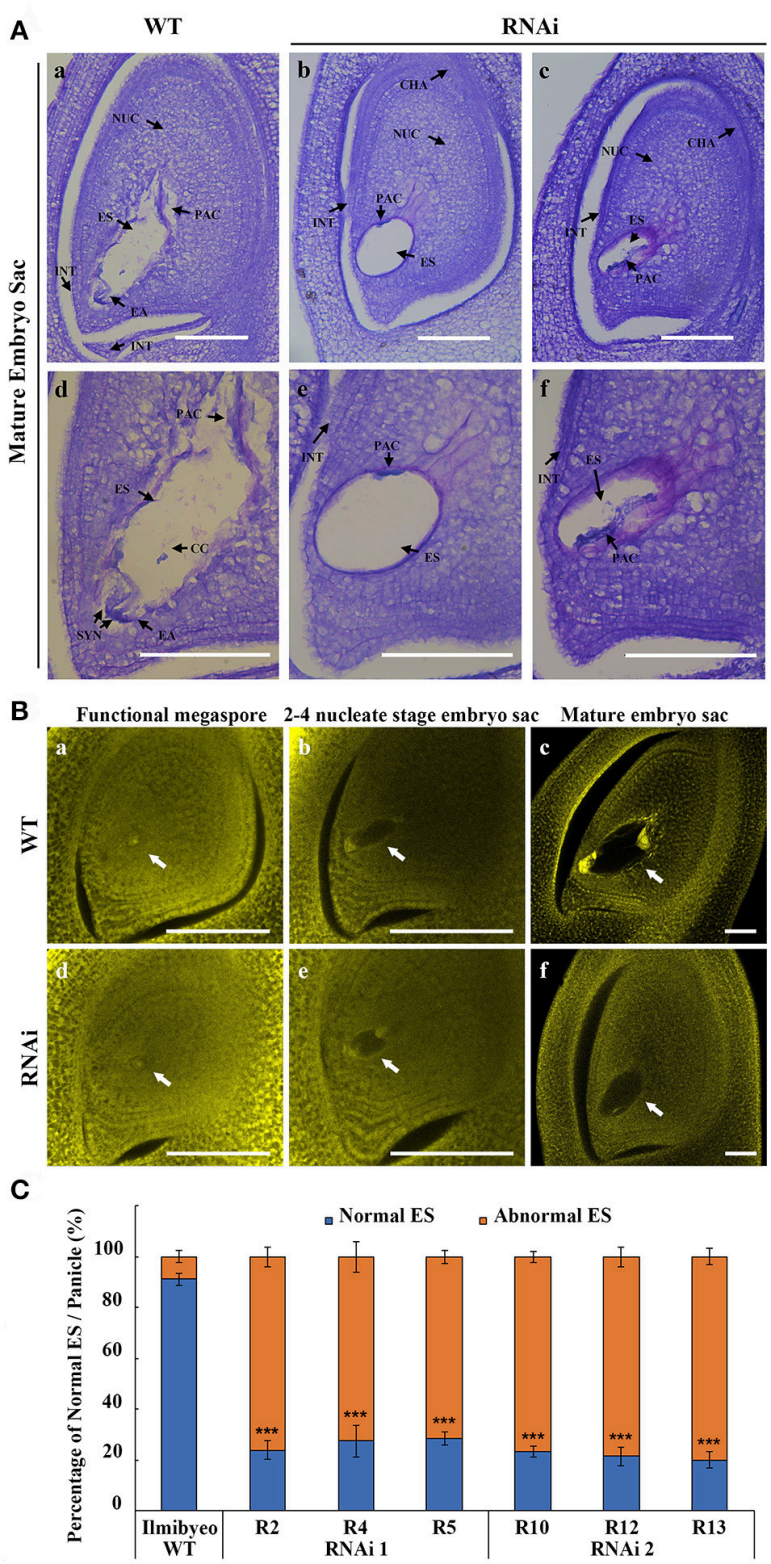
*OsEMSA1* control of embryo sac development. **(A)** Longitude section of embryo sac before flowering stage. **(a,d)** Normal embryo sac in WT plant. (**b**,**e** and **c**,**f**) Different types of undeveloped embryo sacs in *OsEMSA1* RNAi plants. **(d–f)** are enlarged figures of **(a–c)**, respectively. NUC, nucellus; ES, embryo sac; PAC, possible antipodal cell; INT, integument; EA, egg apparatus; CHA, chalaza; SYN, synergid; CC, central cell; Bars = 100 μm. **(B)** Morphology of embryo sac during megagametogenesis phases using confocal microscopy. White arrows respectively indicate **(a,d)** functional megaspores, **(b,e)** 2–4 nucleate stage embryo sacs and **(c,f)** mature embryo sacs in WT and RNAi mutant plants. Bars = 100 μm. **(C)** Percentage of normal embryo sac in WT plants and RNAi plants. Values are mean ± SD. Asterisks indicated extremely significant differences (^***^P < 0.001).

To understand the reason behind the poor seed setting rate, we next examined female gametophyte development in the RNAi transgenic plants. Pistils just before pollination stage were collected from the representative RNAi and WT plants. Embryo sac development in the WT proceeded normally, showing a clear differentiation of antipodal, central cells and an egg apparatus (Figures 5Aa,d). Longitudinal sections of embryo sacs of RNAi plants showed that the gene silencing affected normal development of the embryo sac (Figures 5Ab,e; Figures 5Ac,f; Supplementary Figures 1, 2). RNAi ovary paraffin sections showed various levels of developmental and morphological defects in the embryo sac. Quantitative analysis showed that 80% of the RNAi transgenic plants harbored distorted, irregular and shrunken embryo sacs (Figure 5C). The embryo sacs of RNAi lines were degenerated and with poor differentiation of any gametophytic cells. Observations of the development of embryo sacs in RNAi transgenic plants using confocal microscopy showed that the female gametophyte failed to undergo normal nuclear divisions and cellularization during megagametogenesis (Figure 5B). These results indicated that *OsEMSA1* was involved in embryo sac development in rice.

### *OsEMSA1* affects hormones level in multiple plant tissues

**Figure 6:**
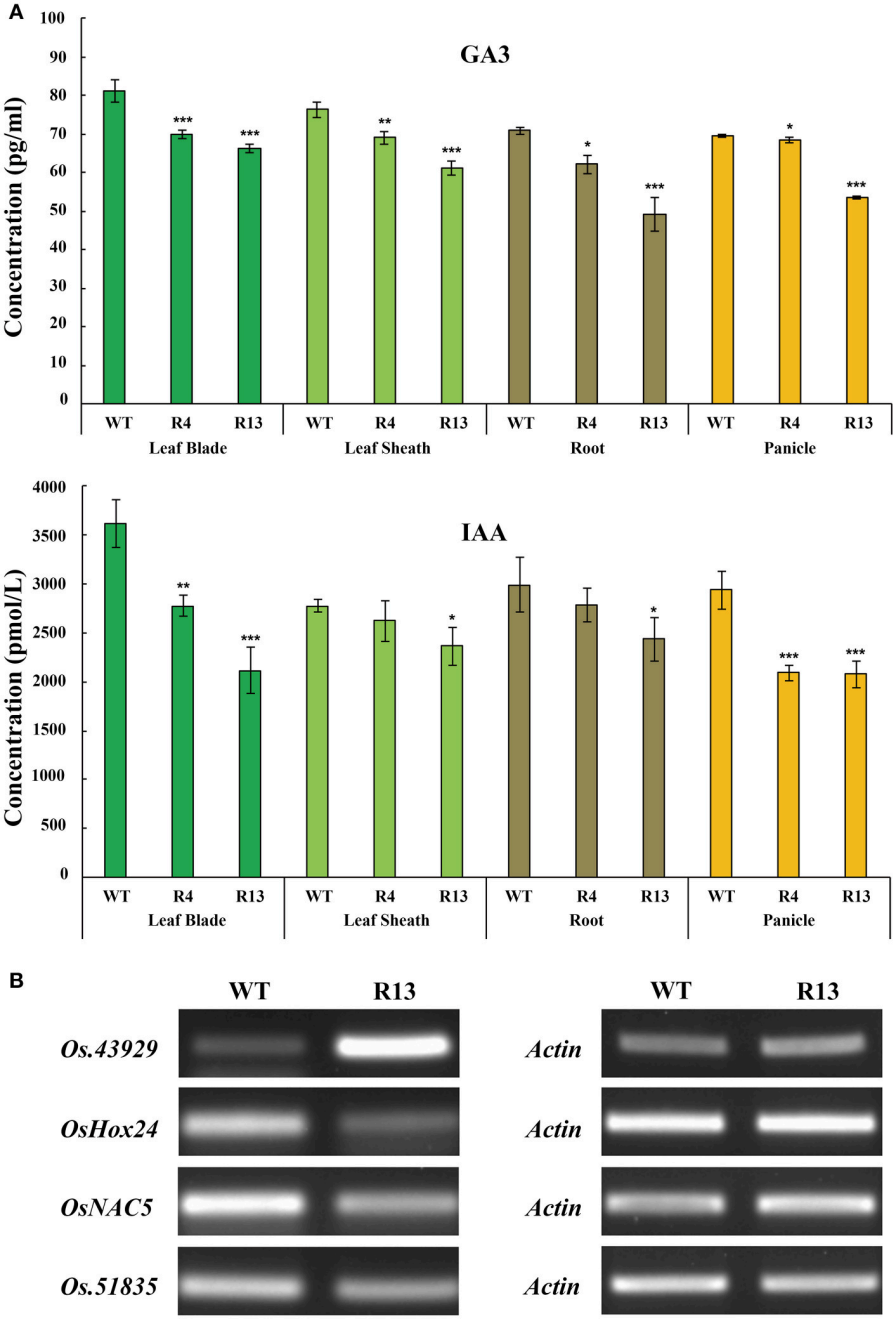
*OsEMSA1* control of hormone distribution and expression of hormone regulator genes. **(A)** Hormone content comparison between WT and *OsEMSA1* RNAi plants at heading stage. Content of GA_3_ and IAA in leaf blade, leaf sheath, root and panicle. Values are mean ± SD. Asterisks indicated significant differences (^*^P < 0.05) and extremely significant differences (^**^P < 0.01 and ^***^P < 0.001). **(B)** RT-PCR analysis of hormone regulator genes expression pattern in *OsEMSA1* RNAi plants.

In our previous study, *fst* mutation decreased the expression of several genes related to developmental and hormonal pathways. *OsEMSA1* is one of the down-regulated genes in the *FST* network. To determine whether this gene had some role in hormone regulation, we explored the hormone contents in different tissues of the transgenic plants. Results indicated that the expression profile of *OsEMSA1* affected the endogenous hormone content in different tissues of the RNAi plants at the heading stage. Gibberellic acid (GA_3_) and indole-3-acetic acid (IAA) content were significantly lower in leaf blade, leaf sheath, roots, and panicle for RNAi lines compared with WT (Figure 6A). We further investigated whether *OsEMSA1* affected expression of genes related to hormone regulation. Four genes which are classified as hormone regulatory genes by gene annotation and which are co-down regulated along with *OsEMSA1*, were selected from our previous microarray data (Lee et al., 2013). *OsHox24, Os.51835, OsNAC5* and *Os.43929* transcript levels were examined in heading stage panicles of *OsEMSA1* RNAi transgenic lines and WT by semi-quantitative RT-PCR (Kikuchi et al., 2000; Yu et al., 2005; Kawahara et al., 2013). The expression level of *Os.43929* was increased, while expression level of the other three genes decreased in the mutant compared with WT (Figure 6B). These results suggested that *OsEMSA1* might have some putative role in hormone regulation in rice.

## Discussion

### Novel function of LysM domain-containing gene *OsEMSA1*

We showed that *OsEMSA1* possessed a conserved C-terminal LysM domain and was involved in development of the embryo sac in rice. Bioinformatic analysis predicted an N-terminal signal peptide, a single LysM domain of 42 amino acid residues and encodes a protein of 108 amino acids. Sequence homology and phylogenetic analysis showed that the protein is widely distributed in both monocotyledons and dicotyledons but with no specific function assigned. Our data on the role of LysM domain-containing gene *OsEMSA1* suggested that this gene may be involved in important functions not previously known. The LysM domain is a widespread protein module and has been found in plants, fungi, animals and humans (Shi et al., 2013). In plants, LysM domain-containing proteins are the second major class of highly conserved pattern recognition proteins and are involved in plant innate immunity (Gust et al., 2012; Shi et al., 2013; Tanaka et al., 2013; Wan, 2015). A typical LysM domain consists of approximately 44–65 amino acids and forms a β-α-α-β-strand secondary structure (Bateman and Bycroft, 2000). In *Arabidopsis*, LysM domain-containing proteins were found to be involved in peptidoglycan and chitin recognition and in turn mediated immunity against pathogen invasion (Miya et al., 2007; Willmann et al., 2011). Mutation in any of the genes significantly compromised *Arabidopsi*s defense responses, leading to increased susceptibility to pathogens (Wan et al., 2008). Rice blast and bacterial blight are the two most devastating rice diseases, and cause significant yield losses around the globe (Liu et al., 2014). Several rice LysM domain-containing proteins directly or indirectly recognize the variable pathogen fragments and trigger defense responses. Silencing these genes considerably increases susceptibility of rice to bacterial and fungal pathogens by blockage of defense mechanisms (Kaku et al., 2006; Shimizu et al., 2010; Liu et al., 2014). In vertebrates, six zebrafish LysM domain-containing genes of two distinct sub-families called *LysMD* and *OXR* were identified and found to be strongly expressed in zebrafish embryos, but none of these genes was responsive to challenge with bacterial pathogens (Laroche et al., 2013). To date, no report on the role of LysM domain-containing proteins in plant sexual reproduction has been presented. In this study, we identified the involvement of *OsEMSA1* in sexual reproduction of rice, which offers a new insight into the functions of LysM domain-containing proteins.

### *OsEMSA1* silencing disrupts embryo sac development in rice

Our results on male gametogenesis indicated that the *OsEMSA1* did not affect anther development. The RNAi transgenic lines produced viable pollen that germinated normally. The growth of the pollen tube toward the pistil indicated that pollen tube growth and sporophytic guidance were similar to the WT. We further investigated development of the embryo sac and observed distinct abnormalities in the embryo sac of the RNAi transgenic plants. Embryo sac development was severely arrested and was much smaller and shrunken with an irregular shape. The embryo sacs were empty and contained no obvious egg apparatus compared with the WT. The embryo sacs of the RNAi lines failed to attract the pollen tubes which after reaching the transmitting tract seemed to lose their path, started abnormal growth patterns and failed to enter the micropylar ending. Successful fertilization requires controlled growth and guidance of the pollen tube until it enters the micropylar opening of the female gametophyte (Palanivelu and Tsukamoto, 2012). Pollen tube guidance requires a complex signaling network that involves gametophytic as well as sporophytic tissues of the female gametophyte (Kasahara et al., 2005; Márton et al., 2005; Chen et al., 2007; Alandete-Saez et al., 2008; Okuda et al., 2009; González-gutiérrez et al., 2014). Mutants with a defective female gametophyte fail to fertilize and develop seeds (Palanivelu and Tsukamoto, 2012). Various studies have mentioned that the embryo sac regulates the micropylar pollen tube guidance. Ling et al. reported the role of SUMO E3 ligase (*SIZ1*) in the functioning of the mature embryo sac in *Arabidopsis*. *SIZ1* mutants developed abnormal embryo sacs, which failed to attract pollen tubes and resulted in decreased seed set (Ling et al., 2012). *Arabidopsis* ovules carrying *magatama3* failed to attract pollen tubes due to delays in embryo sac maturation, indicating that pollen tube guidance signals originated only from mature ovules (Shimizu et al., 2008). *Pollen tube guidance 1* (*PTB1*) is responsible for the sporophytic guidance of pollen tube in rice. *PTB1* encodes a RING-type E3 ubiquitin ligase and is expressed in the stigma and style. *PTB1* is a domestication-related gene and is thought to have been under human selection during rice domestication because it regulates the panicle seed-setting rate (Li et al., 2013). The secreted chemotactic attractants of the female gametophyte have been identified in many plants (Shimizu and Okada, 2000; Higashiyama et al., 2001). However, no study has yet identified any gene in rice involved in gametophytic cell-cell communication. Our study preclude distinguishing between sporophytic vs. gametophytic activity for the RNAi constructs however, the *OsEMSA1* RNAi transgenic plants provide a great opportunity for studying the precise origin of male-female chemotactic signals and the underlying mechanisms involved in sexual reproduction in rice.

To determine whether *OsEMSA1* has some role in hormone regulation in rice, we examined the endogenous content of GA_3_ and IAA in different tissues of RNAi transgenic plants. Results showed that *OsEMSA1* affected the hormone content in different tissues. We further investigated whether *OsEMSA1* had some effect on other genes related to hormone regulation. We selected four genes that were also down regulated in our previous *fst* microarray data (Lee et al., 2013). Gene annotation of our microarray data suggested them to be involved in hormone regulation. Among the four selected genes, the gene *OsHox24* and *OsNAC5* are previously identified to be hormone responsive genes (Sperotto et al., 2009; Bhattacharjee et al., 2016, 2017). *OsHox24* is a member of homeobox transcription factor family and play important role in rice plant growth and development (Bhattacharjee et al., 2017). Previous studies have identified the role of *OsHox24* in abiotic stress responses in rice by regulating the expression of other stress responsive genes (Bhattacharjee et al., 2016, 2017). *OsHox24* has been identified as a hormone responsive gene and is involved in ABA, GA, SA, or IAA-signaling pathway (Olsson et al., 2004; Bhattacharjee et al., 2016). *OsNAC5* is a member of the NAC family transcription factors that regulates abiotic stress responses in rice by modulating the expression of stress-responsive genes (Sperotto et al., 2009; Takasaki et al., 2010; Song et al., 2011). Previous study has identified an ABA- dependent expression of *OsNAC5* during grain filling stage in rice (Sperotto et al., 2009). Our independent experiments on the two co-downregulated genes, *Os.51835* and *Os.43929*, indicated their involvement in hormone regulation in rice (Supplementary Figure 3). Our results indicated that suppression of *Os.51835* and *Os.43929* expression resulted in a reduction of GA_3_ and IAA content in transgenic plants. To investigate the relation of *OsEMSA1* with the other co-downregulated genes, we performed RT-PCR analysis. Our semi-quantitative RT-PCR analysis of RNAi mutant panicles at heading stage showed that *OsEMSA1* also regulated the expression of genes involved in hormone regulation and suggests that *OsEMSA1* was involved in hormone regulation in rice. Phytohormones control different developmental processes in plants. Auxin play important role in ovule patterning in the female gametophyte of *Arabidopsis* (Pagnussat et al., 2009). It was observed that syncitial embryo sac cell fate can be regulated by asymmetrical sporophytic and gametophytic auxin gradient (Pagnussat et al., 2009; Sundaresan and Alandete-Saez, 2010; Panoli et al., 2015). Previous studies showed that auxin source (IAA) is located in the sporophytic tissue at early stage embryo sac then transfer to micropylar end of female gametophyte. The egg apparatus fate would correspond to the highest auxin concentration which is formed in the syncytial embryo sac at the micropylar pole and antipodal fate to the lowest at the chalazal pole (Pagnussat et al., 2007, 2009; Sundaresan and Alandete-Saez, 2010). Although numerous studies have highlighted the role of phytohormones in male and female reproductive organogenesis, but these studies on the embryo sac development in monocots in general and in rice in particular are not comprehensive. Much work needs to be done to explore the molecular mechanisms involved in phytohormones mediated organogenesis. Our present finding supports our previous studies on *fst* mutant in rice whereby the mutant developed defective ovules and complete abortion of the embryo. The precise molecular network of *FST* and its associated genes controlling the various developmental processes in rice is yet to be revealed.

Our results revealed that *OsEMSA1* had a definite role in the development and cellularization of female gametophytic cells in rice. Gene silencing resulted in a defective and degenerated embryo sac that failed to attract pollen tubes. Our results demonstrated that *OsEMSA1* was directly or indirectly play some role in the endogenous hormone regulation and embryo sac development in rice. Our study provides a novel function of LysM domain-containing proteins in female gametophyte development of rice.

## Author contributions

DL and LC conceived the original project and research plans; DL, LC, and CL supervised the experiments; QZ and XZ designed the experiments and performed most of the experiments; QZ, XZ, and SN analyzed the data; SN, WD, XG, and HZ provided technical assistance. SN and QZ wrote the article with contributions of all the authors; All authors supervised and complemented the writing. All authors agree to be accountable for the content of the work.

### Conflict of interest statement

The authors declare that the research was conducted in the absence of any commercial or financial relationships that could be construed as a potential conflict of interest.
