# Supplementary material for: A LysM Domain-Containing Gene OsEMSA1 Involved in Embryo sac Development in Rice (Oryza sativa L.)
Source: Front Plant Sci. 2017 Sep 20;8:1596. doi: 10.3389/fpls.2017.01596 (PMC5611485; doi:10.3389/fpls.2017.01596)
Supplement: Supplementary file 1 [file DataSheet1.docx]

Supplementary Material

A LysM Domain-containing gene *OsEMSA1* involved in embryo sac development in rice (*Oryza sativa* L.)

Qian Zhu, Xiao-Ling Zhang, Li-Juan Chen*, Dong-Sun Lee*

*** Correspondence:** Corresponding Author: 964136487@qq.com; dong_east@ynu.edu.kr

## Supplementary Figures


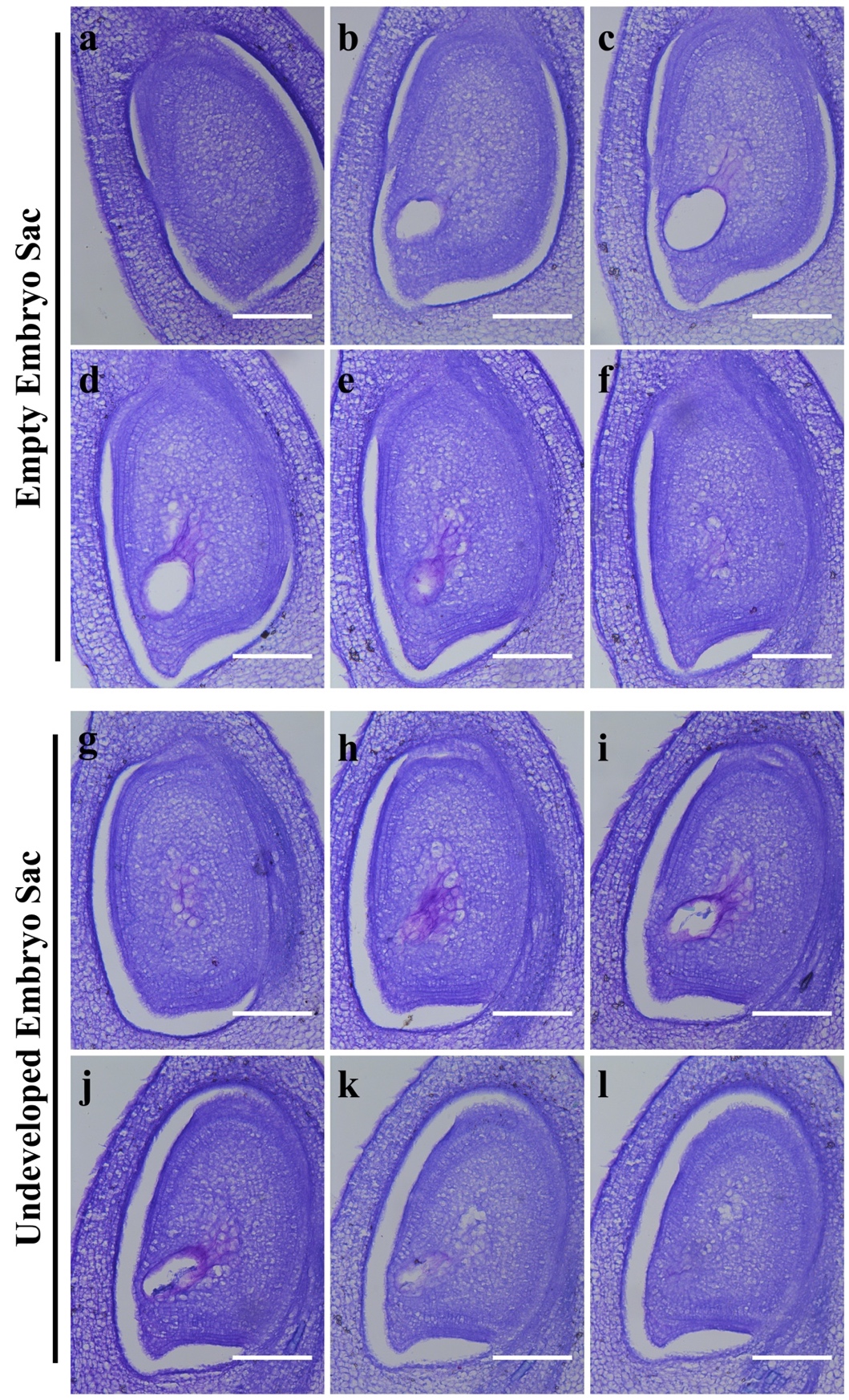


**Supplementary Figure 1.** Series sections of RNAi transgenic embryo sac at before flowering stage. Two different types of transgenic embryo sacs. (a–f) series sections of empty embryo sac. (g–l) series sections of undeveloped embryo sac. Bars = 100 μm.

**
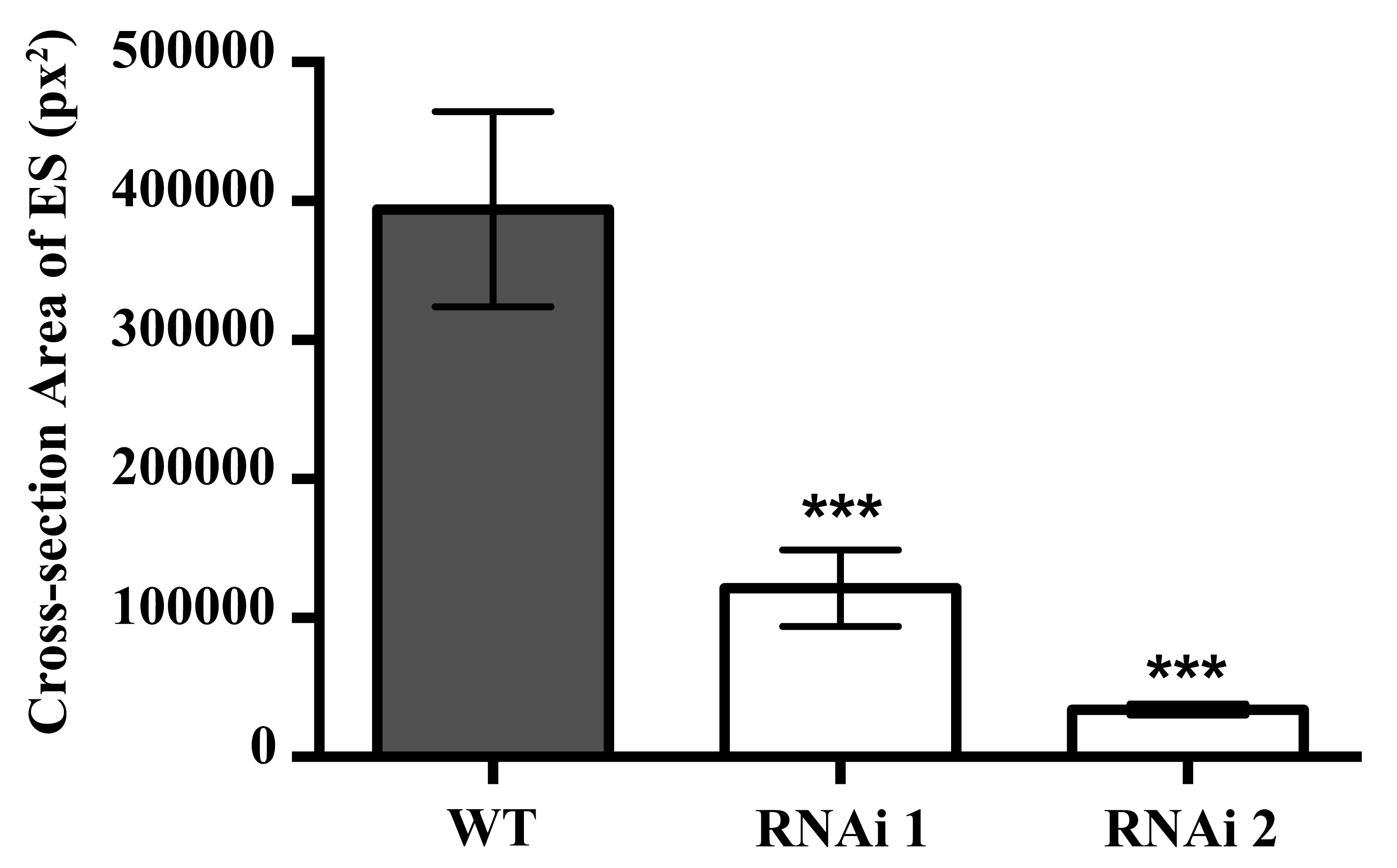
**

**Supplementary Figure 2.** Comparison of embryo sac size in WT and RNAi lines. Values are mean ± SD, asterisks indicated significant differences (* *P* < 0.05) and extremely significant differences (** *P* < 0.01 and *** *P* < 0.001). RNAi1 and RNAi2 are RNAi transgenic lines.


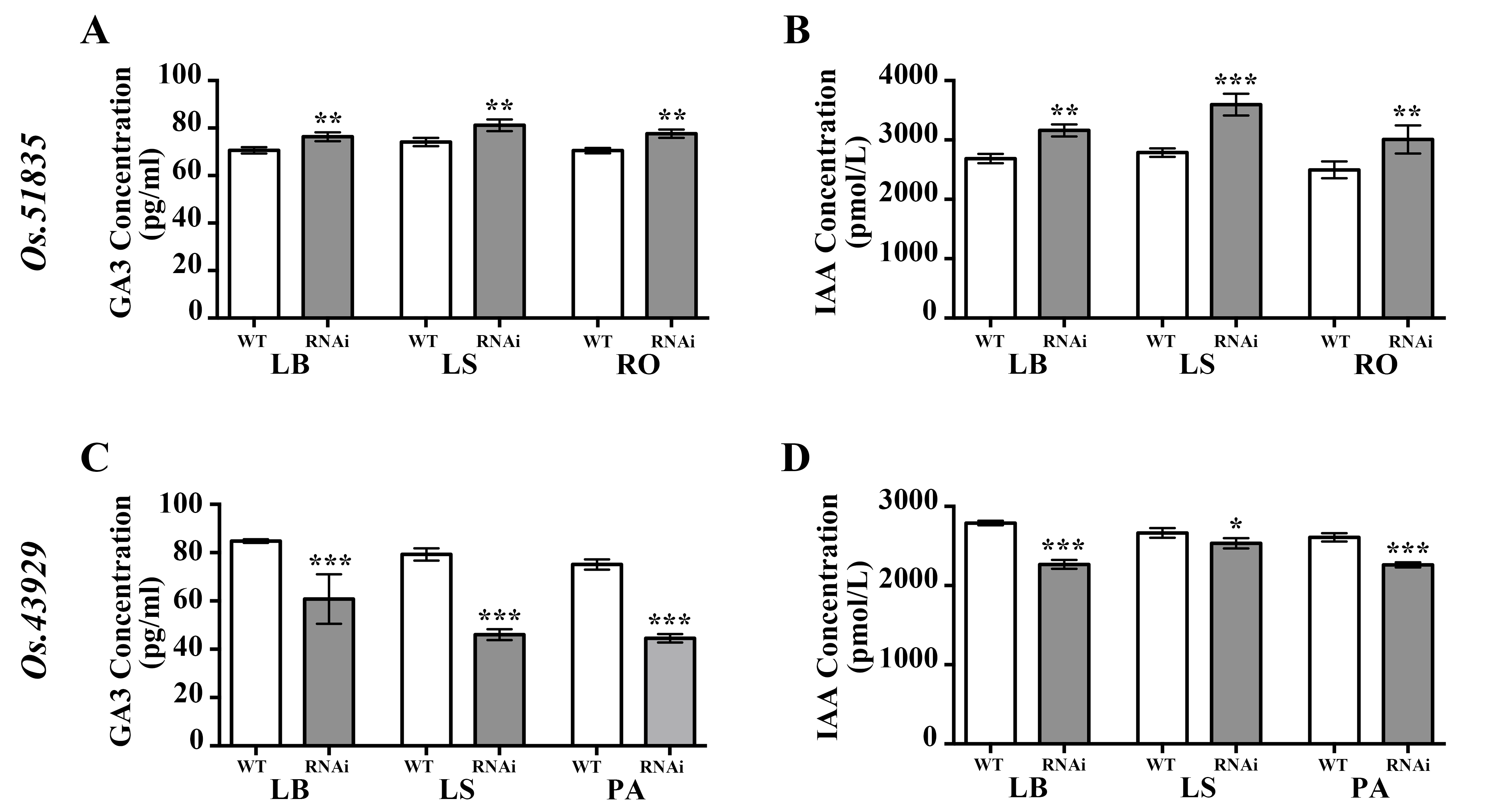


**Supplementary Figure 3.** Quantification of phytohormones in different tissues of *Os.51835* RNAi transgenic plants and *Os.43929* RNAi transgenic plants. (A) Comparison of GA3 contents in WT and *Os.51835* RNAi transgenic plants. (B) Comparison of IAA contents in WT and *Os.51835* RNAi transgenic plants. (C) Comparison of GA3 contents in WT and *Os.43929* RNAi transgenic plants. (D) Comparison of IAA contents in WT and *Os.43929* RNAi transgenic plants. LB, leaf blade; LS, leaf sheath; RO, root; PA, panicle. Values are mean ± SD, asterisks indicated significant differences (* *P* < 0.05) and extremely significant differences (** *P* < 0.01 and *** *P* < 0.001).

## Supplementary Tables

**Supplementary table 1.** *Cis*-acting element prediction of *OsEMSA1* promoter region.

| **Site name** | **Core sequence** | **Position** | **Function** |
| --- | --- | --- | --- |
| **Light Responsiveness** | | | |
| ATCT-motif | AATCTAATCT | -1966 ~ -1958 | part of a conserved DNA module involved in light responsiveness |
| ACE | GCGACGTACC | -1387 ~ -1378 | cis-acting element involved in light responsiveness |
| G-Box | CACGTA | -1808 ~ -1803 | cis-acting regulatory element involved in light responsiveness |
|  | CACGTT | -721 ~ -716 | cis-acting regulatory element involved in light responsiveness |
|  | GTACGTG | -1506 ~ -1500 | cis-acting regulatory element involved in light responsiveness |
|  | GTACGTG | -1386 ~ -1380 | cis-acting regulatory element involved in light responsiveness |
|  | TAAACGTG | -550 ~ -543 | cis-acting regulatory element involved in light responsiveness |
| MRE | AACCTAA | -753 ~ -747 | MYB binding site involved in light responsiveness |
| GAG-motif | AGAGATG | -69 ~ -63 | part of a light responsive element |
|  | AGAGAGT | -16 ~ -10 | part of a light responsive element |
| Sp1 | GGGCGG | -1486 ~ -1481 | light responsive element |
|  | GGGCGG | -1453 ~ -1448 | light responsive element |
|  | GGGCGG | -1417 ~ -1412 | light responsive element |
|  | CC(G/A)CCC | -24 ~ -15 | light responsive element |
| **Hormone Responsiveness** | | | |
| ABRE | CGTACGTGCA | -1507 ~ -1500 | cis-acting element involved in the abscisic acid responsiveness |
|  | ACGTGGC | -1384 ~ -1378 | cis-acting element involved in the abscisic acid responsiveness |
| CGTCA-motif | CGTCA | -1316 ~ -1312 | cis-acting regulatory element involved in the MeJA-responsiveness |
| TGACG-motif | TGACG | -1292 ~ -1288 | cis-acting regulatory element involved in the MeJA-responsiveness |
| TCA-element | CCATCTTTTT | -459 ~ -450 | cis-acting element involved in salicylic acid responsiveness |
| TGA-element | AACGAC | -1729 ~ -1724 | auxin-responsive element |
| GARE-motif | TCTGTTG | -283 ~ -277 | gibberellin-responsive element |
| **Stress Responsiveness** | | | |
| LTR | CCGAAA | -2104 ~ -2099 | cis-acting element involved in low-temperature responsiveness |
| DRE | TACCGACAT | -1365 ~ -1358 | cis-acting element involved in dehydration, low-temp, salt stresses |
| HSE | AAAAAATTTC | -1240 ~ -1231 | cis-acting element involved in heat stress responsiveness |
|  | AAAAAATTTC | -1048 ~ -1039 | cis-acting element involved in heat stress responsiveness |
| MBS | CAACTG | -2051 ~ -2046 | MYB binding site involved in drought-inducibility |
| ARE | TGGTTT | -1844 ~ -1839 | cis-acting regulatory element essential for the anaerobic induction |
| **Growth Regulation** | | | |
| RY-element | CATGCATG | -1742 ~ -1735 | cis-acting regulatory element involved in seed-specific regulation |
| CAT-box | GCCACT | -1664 ~ -1659 | cis-acting regulatory element related to meristem expression |
| Skn-1_motif | GTCAT | -733 ~ -729 | cis-acting regulatory element required for endosperm expression |
| motif I | gGTACGTGGCG | -1387 ~ -1377 | cis-acting regulatory element root specific |
| **Metabolism** | | | |
| O2-site | GATGACATGG | -734 ~ -725 | cis-acting regulatory element involved in zein metabolism regulation |
| **Circadian** | | | |
| circadian | CAANNNNATC | -711 ~ -702 | cis-acting regulatory element involved in circadian control |
| **Transcription** | | | |
| 5UTR Py-rich stretch | TTTCTTCTCT | -200 ~ -191 | cis-acting element conferring high transcription levels |

**Supplementary table 2.** Primer sequences used in the study.

| **Primer name** | **Primer sequence** | **Purpose** |
| --- | --- | --- |
| OsEMSA1-1F | 5′-GGATCCAGCACCAACCAAAACCAAAGCA-3′ | *OsEMSA1* gene-specific primers |
| OsEMSA1-1R | 5′-GTCGACAACAAAGCACACACACACCAGG-3′ |  |
| OsEMSA1P-F | 5′-TCTAGATGCAAAAGCGATTCCGATTTCCG-3′ | *OsEMSA1* promoter-specific primers |
| OsEMSA1P-R | 5′-GGATCCTGCTTTGGTTTTGGTTGGTGCTT-3′ |  |
| OsEMSA1Ri-1F | 5′-TGGCGCTGCTGCTGGT-3′ | *OsEMSA1* RNAi-specific primers/RT-PCR |
| OsEMSA1Ri-1R | 5′-GTTCTGCTCCAGGATGTACGGG-3′ |  |
| OsEMSA1Ri-1F | 5′-TGGCGCTGCTGCTGGT-3′ | *OsEMSA1* RNAi-specific primers |
| OsEMSA1Ri-2R | 5′-GCCCTTCTTTGGTCAGAATCTTTCA-3′ |  |
| OsHox24-1F | 5′-ACGACCATCACCTAGACTACTT-3′ | *OsHox24* gene-specific primers for RT-PCR |
| OsHox24-1R | 5′-TACTGTATGCTACGCTGATTGATT-3′ |  |
| OsNAC5-1F | 5′-TAGCGACTAATCCAGCCTTCGT-3′ | *OsNAC5* gene-specific primers for RT-PCR |
| OsNAC5-1R | 5′-CCACCGGCAGATCAAAATCGAA-3′ |  |
| Os51835Ri-F | 5′-GCGACCGGGACGAGTG-3′ | *Os51835* RNAi-specific primers |
| Os51835Ri-R | 5′-GAATCGGGGTCGGGGG-3′ |  |
| Os51835-1F | 5′-GCTCTGTTTCCTCACTCTGTTGG-3′ | *Os51835* gene-specific primers for RT-PCR |
| Os51835-1R | 5′-CTTGCGGCCCGGCATT-3′ |  |
| Os43929Ri-F | 5′-CTGGCAGGTCAAATGGGAAG-3′ | *Os43929* RNAi-specific primers |
| Os43929Ri-R | 5′-GACCATCTTCCTGAAAGGTAATGA-3′ |  |
| Os43929-1F | 5′-TGCTGTGCCTAAATCAATTATGCCT-3′ | *Os43929* gene-specific primers for RT-PCR |
| Os43929-1R | 5′-GGCATGCACAGAATTGCTGGA-3′ |  |
| ACTIN-F | 5′-CCGAGCGGGAAATTGTGAGGGA-3′ | Actin primers for internal standard of RT-PCR |
| ACTIN-R | 5′-TTTCAGGAGGGGCGACCACCTT-3′ |  |
